# Assessing the quality of hospital outpatient services in Iran: a systematic review and meta-analysis

**DOI:** 10.1186/s12913-023-09506-4

**Published:** 2023-05-18

**Authors:** Pouria Farrokhi, Rafat Bagherzadeh, Morteza Arab-Zozani, Ehsan Zarei

**Affiliations:** 1 Department of Health Management and Economics, School of Public Health, Tehran University of Medical Sciences, Tehran, Iran; 2 English Department, School of Health Management and Information Sciences, Iran University of Medical Sciences, Tehran, Iran; 3 Social Determinants of Health Research Center, Birjand University of Medical Sciences, Birjand, Iran; 4 Department of Health Service Management, School of Management and Medical Education, Shahid Beheshti University of Medical Sciences, Tehran, Iran

**Keywords:** Service quality, Health quality, Outpatient services, Systematic review, Iran

## Abstract

**Background:**

Quality healthcare services are considered one of the most effective vehicles for healthcare managers to achieve organizational goals. Therefore, this study aimed to combine the findings of comparable studies to identify consistencies and contradictions in the quality of outpatient services in Iran.

**Methods:**

The current systematic review and meta-analysis study was conducted in 2022 according to PRISMA guideline. All relevant English and Persian studies were searched in databases, including Web of Sciences, PubMed, Scopus, Scientific Information Database, and Magiran. No year restriction was applied. The quality of the studies was assessed by the 22-item Strengthening the Reporting of Observational Studies in Epidemiology checklist. The meta-analysis was conducted by using Open Meta Analyst, and between-study heterogeneity was investigated with I-squared statistic.

**Results:**

Of the 106 retrieved articles, seven studies with a total sample size of 2600 were included in the meta-analysis. The pooled estimate of mean for overall perception was 3.95 (95% CI: 3.34- 4.55, *P*< 0.001, I^2^= 99.97), while the pooled estimate of the mean for the overall expectation was 4.43 (95% CI: 4.11- 4.75, *P*< 0.001, I^2^= 99.93). The highest and lowest perception mean scores were related to tangibility (3.52, Gap= -0.86) and responsiveness (3.30, Gap= -1.04) dimensions.

**Conclusion:**

Responsiveness was identified as the weakest dimension. Therefore, managers are recommended to design suitable workforce-development programs which focus on the provision of timely and prompt services, polite and courteous interactions with patients, and prioritization of patients’ needs. Moreover, training public sector practitioners along with incentives can fill up the existing gaps.

## Background

In today's competitive environment, the quality of services is one of the most effective vehicles for healthcare managers to achieve organizational goals. High-quality services can increase patient satisfaction and generate loyalty and trust [1, 2]. One of the main challenges of healthcare systems worldwide is the provision of quality services with limited resources. A good healthcare system must balance quality, cost and resource allocation [3]. Specific characteristics of healthcare systems, such as heterogeneity, intangibility, and simultaneity have made the definition of quality more difficult. Crosby defines quality as conformation to specification [4]. In contrast, Deming describes it as services and products designed to satisfy the needs and expectation of customers [5]. Similarly, Parasuraman defines quality as meeting customer expectations [6].

According to the World Health Organization (WHO) health system framework, service quality bridges structural blocks and outcomes. If services are not of sufficient quality, healthcare systems will fail in reaching their final goals, i.e., people’s health [7]. Based on WHO report, between 5.7 and 8.4 million deaths are attributed to poor quality of care in low- and middle-income countries (LMICs) which accounts for approximately 15% of all deaths in these countries. Also, inadequate quality of care costs is estimated $1.4 to $1.6 trillion per year in lost productivity in LMICs [8, 9]. As a lower-middle-income country, Iran has taken significant measures to improve service quality, universal health coverage, and health system's responsiveness. The latest measure is the Health Transformation Plan (HTP) which was first implemented in 2014 to enhance public access to healthcare services and facilities of high quality. Therefore, some measures were taken to improve the quality of outpatient services, increase the number of specialists, and improve hospital facilities and hoteling services [10, 11].

The quality of outpatient services is of great importance because outpatient departments are the first point of contact when patients visit hospitals. They are one of the most important sources of patient flow to hospital inpatient departments; consequently, the manner of service delivery in these departments plays an important role in patients' overall perception of hospital services and their decisions for hospitalization [12]. It is expected that in the future, due to new technologies and shorter waiting time, outpatient departments will have the same or more income for hospitals than inpatient departments [13, 14].

In Iran, the quality of inpatient services and primary health care was investigated in several systematic review studies; however, the quality of outpatient services was neglected [15–17]. Therefore, the present study is the first systematic review and meta-analysis combining the findings of comparable studies to identify consistencies and contradictions in the quality of outpatient services in Iran.

## Methods

The current study was conducted in 2022 according to PRISMA guideline. The processes consist of the following steps; identification process, screening process, eligibility criteria, and selection of articles [18]. In cases where agreement could not be reached, a third reviewer was consulted.

### Identification process

Publications were searched in national and international databases, including Web of Sciences, PubMed, Scopus, Scientific Information Database (SID), and Magiran. Using MeSH headings, we searched for the terms: “service quality”, “quality of services”, “health quality”, “quality of health care”, “outpatient”, “outpatient clinics, hospital”, “ambulatory”, “ambulatory care”, and “Iran”. No year restriction was applied. The full search strategy in PubMed is highlighted in Table 1. These searches were supplemented by screening grey literature sources, including Google Scholar database, relevant reports, and conference abstracts.

**Table 1:** Search strategy used in PubMed, which was adapted to other databases

Database	Set	Strategy		Records (No)
PubMed	#1	MeSH	“Quality of health care”	8,060,831
		Title & Abstract	Service quality” OR “Quality of service” OR “Health quality” OR “Quality of health care”	
	#2	MeSH	“Outpatient clinics, hospital” OR “Ambulatory care”	284,538
		Title & Abstract	Outpatient OR “Outpatient clinics, hospital” OR Ambulatory OR “Ambulatory care”	
	#3	MeSH	Iran	64,713
		Title & Abstract	Iran	
	#4		#1 AND #2 AND #3	404

### Screening process

The study followed PRISMA guidelines for the screening process. The retrieved records were exported to Endnote X8 software, and duplicates were removed. Two reviewers (PF and EZ) independently screened the titles and abstracts to identify relevant studies the full text of which were retrieved for detailed review and data extraction.

### Inclusion criteria

The following criteria were used to select studies: (1) Original articles; (2) Studies on hospital outpatient clinics; (3) Studies reporting the mean scores of service quality dimensions from patients’ viewpoints; (4) The availability of full text articles; and (5) Articles published in English and Persian. Outpatient care is defined as a service or treatment provided by outpatient departments in hospitals (private or public) where the patients are not hospitalized. Therefore, studies on clinical (technical) quality as well as studies conducted in clinics outside hospitals, such as dental clinics, pharmacies, etc., were excluded from the study.

### Study selection

The full texts of all included studies were independently checked by two authors. All eligible or potentially eligible studies were assessed by the third author once again. Additionally, the 22-item Strengthening the Reporting of Observational Studies in Epidemiology (STROBE) checklist [19] was used to assess the quality of the studies. A score between 0 and 7 was considered low quality, 8 and 17 as moderate, and 18 and 22 as high quality. A standard data collection form was used to collect data on author(s), publication year, research design, data collection tool(s), service quality dimensions, as well as the mean scores of the weakest and strongest service quality dimensions.

### Synthesis methods

Between-study heterogeneity was investigated with I-squared (I^2^) statistic. All data related to mean and standard deviation (SD) as effect size were extracted from the included studies and transferred to standard error (SE). The meta-analysis was conducted by using Open Meta Analyst, and random effects model was used to estimate the overall effect size and was expressed as standardized mean differences (SMD) with 95% confidence interval (CI). Meta-analysis was conducted based on the overall perception and overall expectation; moreover, subgroup analyses were performed based on tangibility, reliability, responsiveness, assurance, and empathy dimensions. Due to random effects, equal weights were given to the studies, and the weights were not reported. Similarly, there was no need to report the funnel plot [20] since the number of final studies was less than 10.

## Results

**Fig. 1:**
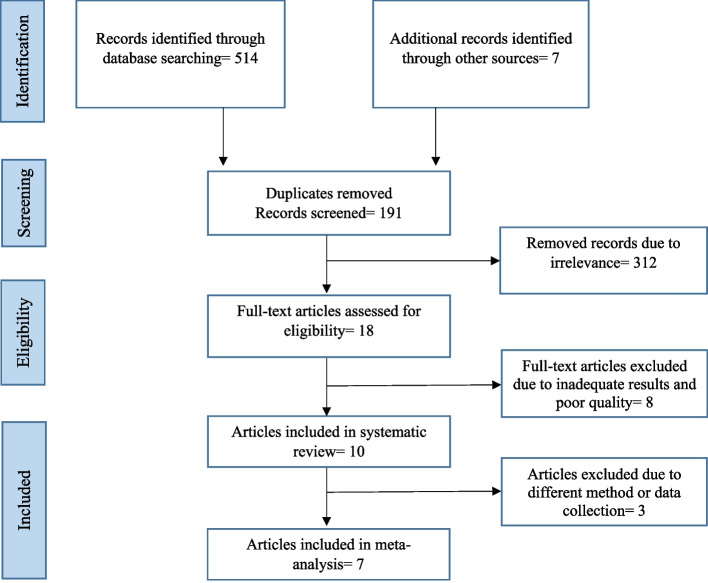
PRISMA flowchart describing the study design

Of the 106 retrieved articles, seven studies met our criteria and were included in the meta-analysis (Fig. 1). The studies were all quantitative and cross-sectional, and a questionnaire, developed by researchers, was used to collect data and the mean scores were measured on a 5-point Likert scale. In 70% of the studies (7 out of 10), the SERVQUAL questionnaire was used for data collection, and 60% of the studies (*n*=6) were conducted in Tehran and published in Persian. The total number of participants was 2600, and the minimum and maximum sample size varied between 200 and 650. The studies were conducted on service quality dimensions, i.e., tangibility, reliability, assurance, responsiveness, empathy, accessibility, physician’s consultation, providing information to patient, physical environment, perceived service costs, appointment, waiting time, and admission process. Eight studies, due to insufficient results and poor quality, were excluded, e.g. Tabibi et al. [21], Abedi et al. [22], and Yavari et al. [23]. In addition, three studies by Zarei et al. [12], Khalili et al. [24], and Abbasi-Moghaddam et al. [14] were disqualified from the synthesis phase because they used various data collection techniques and obtained disparate results.

As indicated in Table 2, 40% of the studies reported the lowest and highest mean scores for responsiveness and tangibility dimensions, respectively. The highest and lowest overall mean scores of patients’ perception were reported in studies by Ghobadi et al. (4.15 ±0.14) [25] and Havasbeigi et al. (2.78 ±0.21) [26], in that order. Furthermore, the largest and smallest gaps between patients’ perceptions and expectations were reported by Farrokhi et al. (gap= -1.55) [27] and, Bastani et al. (gap= -0.57) [28] (Table 3).

**Table 2:** Summary of the selected studies on measuring hospitals’ outpatient service quality

**Author(s)/ Year**	**Location/ Language**	**Sample size/ No of the settings**	**Instrument**	**Dimensions**	**The highest mean score (out of 5)**	**The lowest mean score (out of 5)**
Tabibi et al. 2010 [29]	Tehran/ Persian	242/ 6	SERVQUAL	Tangibility, Reliability, Assurance, Responsiveness, Empathy	Tangibility 3.70	Reliability 3.36
Havasbeigi et al. 2010 [26]	Ilam & Kermanshah/ Persian	450/ NA^a^	SERVQUAL	Tangibility, Reliability, Assurance, Responsiveness, Empathy	Tangibility 3.13	Responsiveness 2.62
Ghobadi et al. 2014 [25]	Ardebil/ Persian	650/ 1	SERVQUAL	Tangibility, Reliability, Assurance, Responsiveness, Empathy	Assurance 4.35	Empathy 3.92
Bastani et al. 2014 [28]	Shiraz/ Persian	200/ 1	SERVQUAL	Tangibility, Reliability, Assurance, Responsiveness, Empathy and Access	Assurance 3.35	Responsiveness 2.78
Khaki et al. 2014 [30]	Shiraz/ Persian	400/ 4	SERVQUAL	Tangibility, Reliability, Assurance, Responsiveness, Empathy	Reliability 3.82	Empathy 3.42
Haghshenas et al. 2015 [31]	Tehran/ Persian	225/ 14	SERVQUAL	Tangibility, Reliability, Assurance, Responsiveness, Empathy	Tangibility 3.72	Responsiveness 3.46
Zarei 2015 [12]	Tehran/ English	500/ 4	Developed by the researchers	Physician’s consultation, Information to patient, Physical environment, Perceived service costs, Appointment, Accessibility, Waiting time, Admission process	Physician’s consultation 4.23	Waiting time 3.10
Khalili et al. 2017 [24]	Tehran/ English	425/ 3	Developed by the researchers	Physical and tangible, Reliability, Accountability, Service assurance, Empathy, Accessibility	Physical and tangible 3.92	Accessibility 3.38
Abbasi-Moghaddam et al. 2019 [14]	Tehran/ English	450/ 4	Developed by the researchers	Accessibility, Appointment, Waiting time, Admission process, Physical environment, Physician’s consultation, Information provision to patient, Service costs	Physician’s consultation 4.17	Waiting time 2.64
Farrokhi et al. 2022 [27]	Tehran/ English	433/ 6	SERVQUAL	Tangibility, Reliability, Assurance, Responsiveness, Empathy	Assurance 3.35	Responsiveness 3.01

^a^Not applicable

**Table 3:** Characteristics of articles included in the meta-analysis

**Article name**	**Dimensions mean score**										**Overall mean score**	
	**Perception**					**Expectation**					**Total**	
	Tangibility	Reliability	Responsiveness	Assurance	Empathy	Tangibility	Reliability	Responsiveness	Assurance	Empathy	Perception	Expectation
Tabibi et al.	3.70 ±0.52	3.36 ±0.43	3.39 ±0.51	3.49 ±0.60	3.44 ±0.61	4.57 ±0.48	4.50 ±0.52	4.59 ±0.58	4.23 ±0.44	4.65 ±0.49	3.47 ±0.29	4.50 ±0.23
Gap (P- E)	-0.87	-1.13	-1.20	-0.74	-1.21						-1.03	
Havasbeigi et al.	3.13 ±0.41	2.79 ±0.36	2.62 ±0.35	2.67 ±0.23	2.72 ±0.34	4 ±0.21	3.95 ±0.28	3.84 ±0.38	4.03 ±0.36	3.86 ±0.39	2.78 ±0.21	3.93 ±0.19
Gap (P- E)	-0.87	-1.16	-1.22	-1.36	-1.14						-1.15	
Ghobadi et al.	3.94± 0.22	4.34 ±0.19	4.19 ±0.18	4.35 ±0.19	3.92 ±0.21	4.79 ±0.17	4.88 ±0.11	4.77 ±0.14	4.79 ±0.19	4.77 ±0.20	4.15 ±0.14	4.80 ±0.11
Gap (P- E)	-0.85	-0.54	-0.57	-0.44	-0.87						-0.65	
Bastani et al.	3.31 ±0.88	3.27 ±0.88	2.78 ±0.92	3.35 ±0.92	2.85 ±1	3.78 ±0.95	3.86 ±0.96	3.57 ±1.17	3.93 ±0.93	3.63 ±0.95	3.61 ±0.68	4.18 ±0.74
Gap (P- E)	-0.47	-0.59	-0.70	-0.58	-0.78						-0.57	
Khaki et al.	3.69 ±0.91	3.82 ±0.94	3.71 ±1.02	3.66 ±0.97	3.42 ±0.95	4.27 ±0.72	4.61 ±0.84	4.53 ±0.71	4.39 ±0.69	4.40 ±0.71	3.66 ±0.71	4.44 ±0.52
Gap (P- E)	-0.58	-0.79	-0.82	-0.73	-0.98						-0.78	
Haghshenas et al.	3.72 ±0.55	3.59 ±0.61	3.46 ±0.66	3.59± 0.66	3.54 ±0.64	4.41 ±0.47	4.43 ±0.48	4.43 ±0.46	4.44 ±0.45	4.34 ±0.48	3.58 ±0.38	4.41 ±0.24
Gap (P- E)	-0.69	-0.83	-0.97	-0.85	-0.81						-0.83	
Farrokhi et al.	3.16 ±0.81	3.23 ±0.90	3.01 ±1	3.35 ±1.23	3.26 ±0.93	4.81 ±0.32	4.80 ±0.39	4.71 ±0.40	4.80 ±0.32	4.68 ±0.47	3.20 ±0.86	4.76 ±0.30
Gap (P- E)	-1.64	-1.56	-1.70	-1.45	-1.42						-1.55	

*P* Perception, *E* Expectation

### Perception

**Fig. 2:**
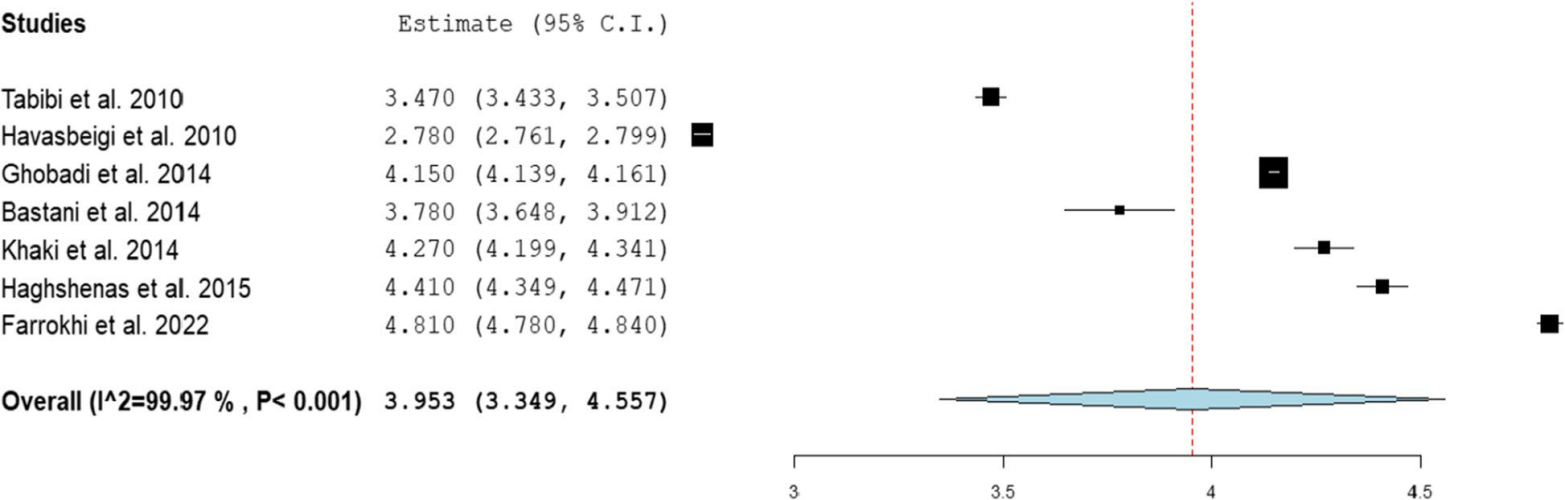
Forest plot for the pooled estimate of the mean for overall perception

There was a high heterogeneity between the studies which can be attributed to different population, setting, gender, and age of the participants. According to the random effect model, the pooled estimate of mean for overall perception was 3.95 (95% CI: 3.34- 4.55, *P*< 0.001, I^2^= 99.97; see Fig 2). Also, regarding the dimensions, the pooled estimate of mean was 3.49 (95% CI: 2.71- 4.28, *P*< 0.001) for the assurance dimension, 3.31 (95% CI: 2.79- 3.82, *P*< 0.001) for the empathy dimension, 3.49 (95% CI: 2.84- 4.13, *P*< 0.001) for the reliability dimension, 3.30 (95% CI: 2.63- 3.99, *P*< 0.001 for the responsiveness dimension, and 3.52 (95% CI: 3.19- 3.85, *P*< 0.001) for the tangibility dimension (Table 4).

**Table 4:** Subgroup analysis based on the dimensions

	Dimension	Number of studies	Mean	Gap P-E	SE	95% CI		Heterogeneity	
						Lower	Upper	I^2^	*P*-value
**Perception**	Tangibility	7	3.52	-0.86	0.167	3.195	3.849	99.96	*P*< 0.001
	Reliability	7	3.49	-0.94	0.330	2.840	4.132	99.92	*P*< 0.001
	Responsiveness	7	3.30	-1.04	0.347	2.628	3.989	99.93	*P*< 0.001
	Assurance	7	3.49	-0.88	0.400	2.711	4.278	99.96	*P*< 0.001
	Empathy	7	3.31	-1.02	0.262	2.795	3.820	99.66	*P*< 0.001
**Expectation**	Tangibility	7	4.38	-	0.170	4.04	4.71	99.87	*P*< 0.001
	Reliability	7	4.43	-	0.188	4.06	4.80	99.87	*P*< 0.001
	Responsiveness	7	4.35	-	0.163	4.03	4.67	99.78	*P*< 0.001
	Assurance	7	4.37	-	0.138	4.10	4.64	99.73	*P*< 0.001
	Empathy	7	4.33	-	0.166	4.01	4.65	99.75	*P*< 0.001

### Expectation

**Fig. 3:**
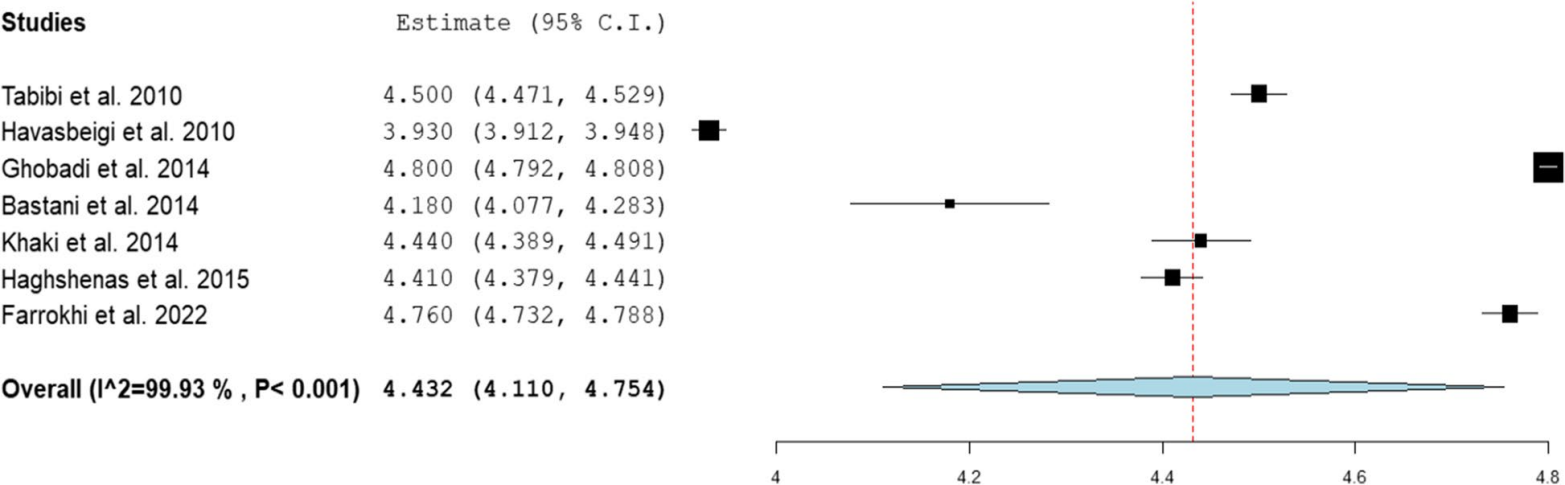
Forest plot for the pooled estimate of the mean for overall expectation

According to the random effect model, the pooled estimate of the mean for the overall expectation was 4.43 (95% CI: 4.11- 4.75, *P*< 0.001, I^2^= 99.93; see Fig 3). Also, regarding the dimensions, the pooled estimate of mean was 4.37 (95% CI: 4.10- 4.64, *P*< 0.001) for the assurance dimension, 4.33 (95% CI: 4.01- 4.66, *P*< 0.001) for the empathy dimension, 4.43 (95% CI: 4.07- 4.80, *P*< 0.001) for the reliability dimension, 4.35 (95% CI: 4.03- 4.67, *P*< 0.001) for the responsiveness dimension, and 4.38 (95% CI: 4.04- 4.71, *P*< 0.001) for the tangibility dimension (Table 4).

The pooled estimate of the mean scores for perception and expectation was calculated: the overall gap between them was -0.48 which was equal to -0.88, -1.02, -0.94, -1.04, and -0.86 for assurance, empathy, reliability, responsiveness, and tangibility dimensions, respectively (Table 4).

## Discussion

For the first time, this study aimed to systematically review the available evidence on outpatient service quality in Iran. The overall mean score of outpatients’ perception (3.95) and expectation (4.43) about the quality of outpatient services illustrates a negative quality gap (-0.48) indicating that patients do not receive expected services, in other words, there is a gap between their expectations and the provided services, and their expectations are not sufficiently met. These results are consistent with Rezaei et al.’s study [15] which indicated that the overall mean scores of patients’ perception, expectation, and the gap between them were 3.69, 4.59, and -0.9, respectively. Similarly, in other studies by Teshnizi et al. [16] and Gilavand & Torabipour [32], the overall service quality gaps in health care centers were -1.64 and -0.86, in that order. Moreover, the overall service quality gap in primary healthcare services was found to be -0.83 by Gorji et al. [17] and -0.53 by Rahmani et al. [33]. Totally, outpatient service quality in Iran is in a good condition which can be attributed to factors, such as shorter length of stay, low out-of-pocket payment, and short-term treatment results.

The lowest overall perception mean score (3.30) was found to be related to the responsiveness dimension which obtained the lowest mean scores in four studies (40%). Responsiveness refers to organizational readiness to help patients, the behavior and attitude of the staff, waiting time and the provision of prompt services. It seems that the appointment and timely service delivery processes in outpatient centers were not satisfactory due to overcrowding, weak motivators, and lack of training of employees in answering patients' questions [27, 28, 31]. Similarly, a study in Turkey reported responsiveness and empathy as the lowest perceived dimensions (5.7 out of 9) [1]. In contrast, the results of a study in private hospitals in Syria showed the highest mean score (4.17) for responsiveness [34].

The highest overall perception mean score was related to the tangibility (3.52) dimension, i.e., organizations’ physical facilities, equipment, and the appearance of the personnel. According to the results, the patients were more satisfied with the tangibility dimension. In accordance with these findings, previous study by Qolipour et al. on service quality of medical tourism in private and public hospitals demonstrated that the tangibility dimension obtained the highest perception mean score and the lowest quality gap (3.92, Gap= -0.68) [35].

Furthermore, the highest and lowest quality gaps were related to the dimensions of responsiveness (-1.04) and tangibility (-0.86) which corroborate the findings of previous works [32, 36, 37] in which the highest quality gap was related to the responsiveness dimension. Therefore, it seems that adequate number of professional human resources and reduction in patient waiting time can help the improvement of this dimension. A systematic review by Batbaatar et al. showed that patients who had to wait longer in the outpatient department, without prior notice, tended to be less satisfied with the overall services [38]. In contrast, a study on the quality of outpatient services in Saudi Arabia indicated the highest gap (-1.42) in the tangibility dimension [39].

## Study limitations

However, these findings are subject to publication bias, a problem that can distort the obtained estimations. Publication bias arises from aspects, such as language bias, multiple publications, selective outcome reporting, poor methodological design, and inadequate data analysis. The insufficient number of studies did not allow us to perform further analysis, such as funnel plots.

## Conclusion

According to the results, few studies have been conducted on outpatient service quality which requires further attention by researchers. Moreover, it is suggested that researchers simultaneously measure the quality of outpatient, inpatient and primary care to understand why patient are more satisfied with outpatient services. The results of the current study can be used to better identify the strengths and weaknesses of outpatient services rendered by health organizations in Iran. Therefore, it is recommended that managers design suitable workforce-development programs focusing on the provision of timely and prompt services, polite and courteous interactions with patients, and prioritization of patients’ needs. Additionally, training public sector practitioners along with incentives can fill up the existing gaps.

## Data Availability

The datasets used and analyzed during the current study available from the corresponding author on reasonable request.

## Abbreviations

WHO: World Health Organization
LMICs: Low- and middle-income countries
HTTP: Health Transformation Plan
STROBE: Strengthening the Reporting of Observational Studies in Epidemiology
I^2^: I-squared
SD: Standard deviation
SE: Standard error
SMD: Standardized mean differences
CI: Confidence interval
